# Around the Hospitals

**Published:** 1893-01-21

**Authors:** 


### AROUND THE HOSPITALS.

The **Royal Albert Edward Infirmary, Wigan,** is very evidently becoming one of those institutions looked upon especially as their own by the working classes. It has, like many another institution, had to pass through a period when success was by no means an assured prospect; but the hospital has now gained the confidence of the neighbourhood, which will doubtless ensure for it a prosperous career henceforward. The past year has been especially marked by tokens of popularity in the large numbers of visits made to it by representative bodies in the neighbourhood, and the institution—which already obtains a larger income from the working classes than any other infirmary in England—may expect still further support from this source than has been afforded heretofore. No increase in the number of beds available has been made during the present year, but most necessary additions in other respects have been made. Twenty-four nurses and servants' bed-rooms have been added, the laundry has been rebuilt, and a steam-boiler and all modern machinery introduced. The drainage has been reconstructed throughout the hospital, and the operating theatre and wards adjoining have been enlarged, and a day room and entertainment-room for the patients has been extended also. All this has been effected at a cost of £5,545. The expenditure of this sum has put the hospital in efficient working order, the additions being much needed, as the administration department had not been added to since the institution was first erected for the treatment of thirty patients only. The ambulance service has been extended, and now serves the Leigh district. The rules of the infirmary provide for the self-respect of the patients, by requiring suitable fees from those whose income exceed certain specified amounts.

